# Draft Genome Sequence of *Burkholderia cenocepacia* Strain 869T2, a Plant-Beneficial Endophytic Bacterium

**DOI:** 10.1128/genomeA.01327-15

**Published:** 2015-11-12

**Authors:** Ying-Ning Ho, Chieh-Chen Huang

**Affiliations:** a Department of Life Sciences, National Chung Hsing University, Taichung, Taiwan; b Agricultural Biotechnology Research Center, Academia Sinica, Taipei, Taiwan

## Abstract

An endophytic bacterium, *Burkholderia cenocepacia* 869T2, isolated from vetiver grass, has shown its abilities for both *in planta* biocontrol and plant growth promotion. Its draft genome sequence was determined to provide insights into those metabolic pathways involved in plant-beneficial activity. This is the first genome report for endophytic *B. cenocepacia*.

## GENOME ANNOUNCEMENT

*Burkholderia* encompasses a wide range of species, including opportunistic human and plant pathogens and symbiotic species that can form beneficial plant-microbe interactions (1). Although recent phylogenetic analyses of *Burkholderia* species distinguished those plant symbionts from opportunistic pathogens and separated into two main clusters (2, 3), there is still considerably tremendous genetic variation that exists among species of these two clusters, and more strain-based genomic informatics is needed to distinguish their phenotypic features.

An endophytic bacterium, *Burkholderia cenocepacia* strain 869T2, has shown its beneficial abilities for *in planta* biocontrol of *Fusarium* wilt of banana (4). The study showed that the disease incidence of *Fusarium* wilt on 869T2-inoculated banana plants (Cavendish cv. Pei-Chiao) was 3.4%, compared to 24.5% of noninoculated plants infected in the field test. Furthermore, a significant plant growth-promoting effect was observed, while 869T2-inoculated banana plants growth in the field (4). Although the *B. cenocepacia* species has been considered the causal agent of banana fingertip rot (5), strain 869T2 never caused any symptoms of the disease, such as a small and externally distorted shape of affected banana fruit fingers. As there is no endophytic bacterium *B. cenocepacia* genome that has been sequenced before, the genomic data of 869T2 may offer the possibility for comparative analysis within related *B. cenocepacia* genomes and provide more insights into specific traits.

DNA was isolated using the Qiagen DNA purification kit, according to the manufacturer’s instructions. Indexed adapters were then ligated to the DNA fragments by DNA ligase, followed by performing PCR to enrich the adapter-modified DNA fragments. After validating the libraries by quantitative PCR (qPCR), Expression, and Qubit, the library was sequenced using Illumina HiSeq 2500. The draft genome sequence of 869T2 comprises 7,979,445 bases, 29,080,976 reads, 84 contigs, and a high G**+**C content of 67.1%. A soil-associated bacterium, *B. cenocepacia* MC0-3, was used as a reference sequence (accession no. GCA_000019505.1). We annotated the predicted protein sequences by conducting a BLASTp search against the reference genome. Unique genes in the assembled genome were found by doing another BLASTp search against the NCBI nr protein database (release February 2013). About 90% of the open reading frames (ORFs) have orthologs in the reference strain MC0-3, while 505 ORFs were unique genes. Of these previously unreported ORFs, 155 ORFs did not generate hits in current public databases (NCBI nr database).

The genome of 869T2 was found to contain genes related to pyrrolnitrin synthesis, which is a common broad-spectrum antifungal agent (6). The annotations of the draft genome sequence also contained 1-aminocyclopropane-1-carboxylate (ACC) deaminase, pyrroloquinoline quinone (PQQ), which are the factors involved with plant growth promotion (7), and dioxin degradation-related genes (2-oxopent-4-enoate hydratase). Analysis of the genome of 869T2 will help us elucidate the metabolic pathways that are involved in the production of antifungal and plant growth-promoting compounds.

### Nucleotide sequence accession numbers.

The whole-genome shotgun (WGS) project of *B. cenocepacia* 869T2 was deposited at DDBJ/EMBL/GenBank under the WGS project accession no. JJOA00000000. The version described in this paper is the first version, JJOA00000000.1.
